# CO_2_ hydrate formation kinetics based on a chemical affinity model in the presence of GO and SDS

**DOI:** 10.1039/c9ra10073c

**Published:** 2020-04-01

**Authors:** Lijun Li, Shuhua Zhao, Shuli Wang, Yongchao Rao

**Affiliations:** School of Petroleum Engineering, Changzhou University Jiangsu 213164 China wsl@cczu.edu.cn

## Abstract

Hydrate generation promotion and kinetic models are key issues in the hydrate utilization technology. The formation kinetics of CO_2_ hydrates in a graphene oxide (GO) and sodium dodecyl sulfate (SDS) compounding accelerator system was studied experimentally, and the influences of different concentrations on the hydrate formation time and gas consumption were revealed. The results show that with the combination of GO and SDS, the formation rate of CO_2_ hydrates was accelerated, the induction time and generation time were shortened, and the gas consumption increased. The optimal compounding concentration was 0.005% GO and 0.2% SDS. Compared with the observations for pure water and a single 0.005% GO system, the hydrate formation time was shortened by 69.7% and 12.2%, respectively, and the gas consumption increased by 11.24% and 3.2%. A chemical affinity model of CO_2_ hydrate formation was established for this system. The effects of the GO and SDS compound ratio, temperature and pressure on the chemical affinity model parameters were studied from the model point of view. On using Matlab to program the model and compare it with the experimental results, very good agreement was achieved. Through research, the chemical affinity model can accurately predict the formation of hydrates in complex systems.

## Abstract 2

Hydrate generation promotion and kinetic models are key issues in the hydrate utilization technology.

## Graphical abstract

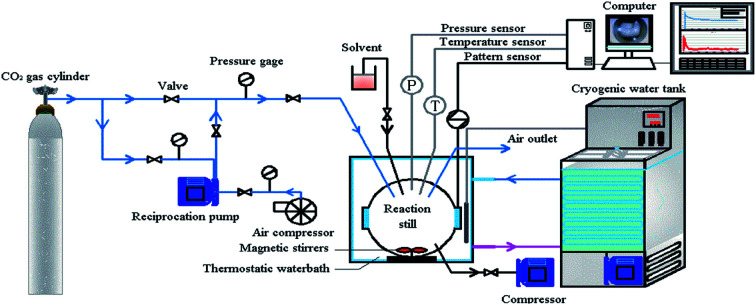

## 1 Introduction

Gas hydrates are non-stoichiometric ice clathrates^1^ formed by host molecules (water) and guest molecules (gas molecules) at a low temperature and high pressure. Usually, 1 cubic meter of hydrates can decompose 160–180 cubic meters of gas.^2^ Due to the high gas storage characteristics of hydrates and the different temperature and pressure equilibrium conditions of different gas-forming hydrates, they have broad application prospects in natural gas storage and transportation,^3,4^ refrigeration technology,^5^ seawater desalination,^6^ gas separation,^7^*etc.* The key to this technology is how to reduce the generation conditions, increase the generation speed by various means, and establish the corresponding dynamics calculation model.

At present, the methods for enhancing the formation of gas hydrates are mainly classified into physical strengthening and chemical strengthening. The physical strengthening method involves increasing the contact area of gas–liquid, enhancing the mass transfer and heat transfer to realize the rapid formation of gas hydrates.^8^ Common methods include the stirring method,^9^ bubbling method,^10^ spray method^11^ and field method.^12^ The chemical strengthening method involves promoting the formation of hydrates by adding a promoter to reduce the surface tension and change the microstructure.^13,14^ Hydrate accelerators are divided into thermodynamic accelerators and kinetic accelerators. The most widely used accelerators include tetrahydrofuran (THF),^15^ sodium dodecyl sulfate (SDS),^16^ sodium dodecyl benzene sulfonate (SDBS),^17^ and tetrabutyl ammonium bromide (TBAB).^18^ Studies have shown that SDS^19^ is an effective additive to promote the rapid formation of gas hydrates. Scholars have also researched the formation characteristics of gas hydrates by nanoparticles such as Al_2_O_3_, CuO, SiO_2_ and carbon nanotubes.^20–23^ Yan S.^24^ studied the effects of solvents containing graphene oxide (GO for short) nanoparticles on the formation of CO_2_ hydrates. The experimental results showed that GO can enhance nucleation efficiency and heat and mass transfer efficiency, shorten the induction time, increase gas storage capacity, and reduce equilibrium pressure; it is also non-toxic and harmless to the environment due to its unique microstructure and properties. In addition, researchers^25^ complexed nano-graphite particles and SDS and found that the compounding system could shorten the induction time and accelerate the nucleation rate but did not give a kinetic model.

At present, common methods for predicting changes in the temperature–pressure and other parameters during hydrate formation include the empirical formula method, phase equilibrium calculation method, statistical thermodynamic model method and graphic method. According to different entry points of hydrate formation conditions and research problems, many models have been established, such as the heat and mass transfer model,^26^ gas consumption rate semi-empirical model^27^ and so on. However, due to the randomness and complexity of the hydrate formation process, especially in systems with additives, the existing models cannot be applied to various working conditions and some parameters are not easy to measure. As a result, there are large model errors and the formation of hydrate cannot be accurately predicted.

Chemical affinity is the general driving force for chemical reactions. All chemical reactions proceed toward a decrease in chemical affinity. At the end of the reaction, the chemical affinity is zero. The model is based on the principle of thermodynamics and can be used to predict the rate of hydrate formation. It is characterized by a relatively simple and easy solution and does not require difficult parameters such as heat transfer and mass transfer coefficients and so on. The rate of hydrate formation can be predicted as long as macroscopic data such as system temperature and pressure and so on, are available. The model has attracted the attention of many researchers. Varaminian *et al.*^28,29^ used the chemical affinity model to study the kinetics of hydrate formation in different systems. The calculated results agreed well with the experimental results.

In 2019, Khurana *et al.*^30^ undertook CH_4_–THF uptake modelling by first performing thermodynamic modelling. The model was then validated for CH_4_–THF and CO_2_–THF systems with varying THF concentrations. They proposed a 2 step CH_4_–THF hydrate growth model and a mass transfer-based model. Insights from the model were discussed and the optimum reactor configuration for SNG application was obtained. Dashti *et al.*^31^ presented a new variation of the shrinking core model (SCM) and a model-based estimation of the induction time. The proposed concept is generic enough to be used for the CH_4_ hydration process too. In 2020, Zhang *et al.*^32^ employed the Magnetic Resonance Imaging technique to analyze the hydrate growth micro-processes for greenhouse gases (CO_2_, CH_4_, and various fractions of CO_2_–CH_4_ mixed gases) and volatile organic compound (simulated by C_2_H_4_ and C_2_H_2_ gases) capture and storage. A multi-pressure control mechanism for secondary hydrate growth was developed to promote CO_2_ capture and storage.

SDS is a well-studied and effective hydrate kinetic accelerator. GO has unique microstructure and surface functional groups, and it can promote both thermodynamics and kinetics of hydrate. In this paper, through the combination of theoretical analysis and experimental research, the characteristics of CO_2_ hydrate formation in the complex system of GO and SDS were studied, and the effects of concentration, temperature and the pressure of the complex accelerator on the formation of CO_2_ hydrate were investigated. The change in gas consumption with time in the process of the formation and growth of CO_2_ hydrate crystals was analyzed by using the chemical affinity model, which provides theoretical guidance for the development of gas hydrate formation promotion technology.

## 2 Experiments

### 2.1 Experimental device

**Fig. 1:**
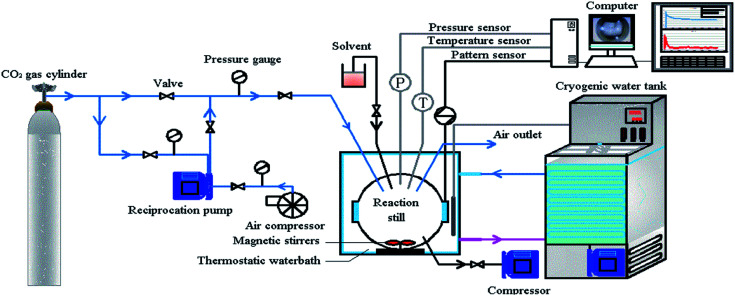
Schematic diagram of the experimental device.

An electronic scale (Shanghai Yueping Scientific Instrument Co. LTD.), with standard deviation of ±0.0002 g and model number FA2104B, was used to weigh the experimental materials, and an ultrasonic processor (Shanghai Shengxi Ultrasonic Instrument Co. LTD.) with model number FS-1200N was used to conduct ultrasonic oscillation on large GO particles and SDS nanomaterials. The experiment was carried out by using a high-pressure magnetic power stirring hydrate-generating device, which was mainly comprised of a high-pressure gas supply system, an air intake system, a high-pressure reactor generating device, a constant temperature cooling water tank and a circulating water bath device, a fiber optic camera system and a data acquisition system. The CO_2_ gas was injected into the high-pressure reactor from the high pressure gas cylinder through the pressure pump and the air compressor. The spherical high-pressure reaction kettle was the main reaction device for the hydrate formation. The design pressure was 30 MPa, the design temperature was 0–20 °C, and the visual kettle volume was 500 ml. The constant temperature refrigerating water tank was filled with a solution of ethylene glycol and water in a 3 : 1 ratio. The circulating water bath device (THD-2030 type) had a temperature control range of −15–20 °C. The temperature control accuracy was ±0.1 °C; the fiber optic camera system was capable of observing and recording the changes in the kettle and the hydrate formation process; the data acquisition system mainly recorded the pressure and temperature changes and collected data through the Agilent 34972a data acquisition instrument. The hydrate formation experimental device is shown in Fig. 1.

### 2.2 Experimental materials

The specifications of the experimental materials are shown in Table 1.

**Table 1:** Specifications of experimental materials

Component	Purity	Supplier
Carbon dioxide	≥99.8%	Jinghua Co. LTD (China)
Distilled water	≥99.9%	Changzhou University (China)
Sodium dodecyl sulfate	≥99%	Sihewei Chemical Co. LTD (China)
GO	≥99%	Jiangnan Graphene Institute (China)

### 2.3 Experimental steps

#### 2.3.1 Configure reagents

The mass fractions were 0.003%, 0.005%, 0.01%, 0.02% for GO and 0.005%, 0.1%, 0.2%, and 0.3% for SDS. According to the experimental requirements, appropriate amounts of GO and SDS were weighed in an electronic balance and placed in a beaker, then 170 ml of distilled water was added and the mixture was subjected to ultrasonic vibration for 20 to 30 minutes to obtain a uniformly dispersed compounding reagent.

#### 2.3.2 Experimental preparation

Before each experiment, the reactor was washed three times with distilled water and then rinsed once with a compounding reagent. The vacuum pump was opened and the reaction kettle and the pipeline were vacuumed for 4–5 min until the pressure in the autoclave reached about −0.1 MPa. The reagent was sucked into the kettle by vacuum and pumping was continued for 4–5 min for the extraction of gases from the kettle and solution.

#### 2.3.3 Generate an experiment

The low-temperature water-bath system was turned on and the experimental temperature value was set and allowed to stabilize for 20–30 min. The CO_2_ cylinder valve was opened slightly and the kettle was slowly pressurized with the booster pump and air compressor. After the intake, the magnetic stirrer was turned on to increase the gas–liquid contact area, and the formation of CO_2_ hydrate was accelerated. The equilibrium conditions of the hydrate formation phase were determined by the constant temperature pressure search method.^33^ The formation of hydrate in the autoclave was observed using a fiber-optic camera, and the temperature, pressure change, and reaction time were recorded by a data acquisition instrument. Since the formation of hydrate is a random process, the data obtained from the experiment are the average values obtained after multiple experiments.

## 3 Chemical affinity model

For a chemical reaction, the chemical affinity *A*_i_ of the i state as the driving force in the reaction is expressed as follows:

1 *A*_i_ = −*RT* ln(*ζ*_*Q*_i__)

In which2
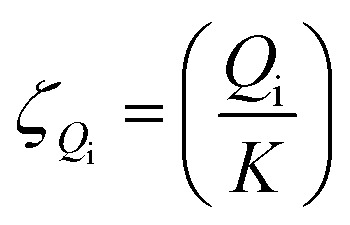


Definition of *Q*_i_ for reaction activity:3
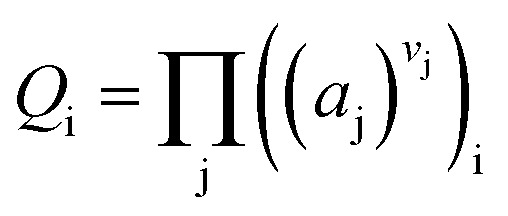
*a*_j_ represents the activity of the components; j, *V*_j_ represents the stoichiometric coefficient of the component j. *R* is the molar gas constant; *T* is the system temperature; *K* represents the thermodynamic equilibrium constant. The reaction activity when the equilibrium state is reached is given by *A*_i_ = 0, therefore. *A*^0^_i_ = *RT* ln(*K*).

*ζ*_*Q*_i__ indicates the extent to which the reaction is close to equilibrium.

For a closed, fixed volume and constant temperature system, the affinity decay rate can be expressed as follows:4
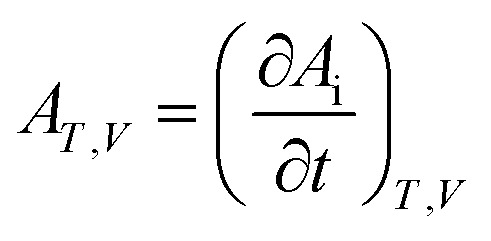


Many related research results have shown that *A*_*T*,*V*_ is inversely proportional to the time, so the following expression can be written for the affinity decay rate:5
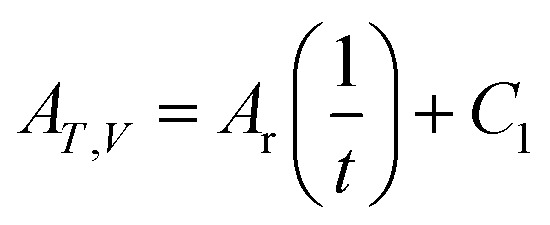
in which *A*_r_ is the proportionality constant and denotes the affinity rate constant. By defining the time needed for the system to reach its equilibrium condition as *t*_k_, when the reaction reaches the equilibrium state, the affinity decay rate *A*_*T*,*V*_ = 0, which gives6
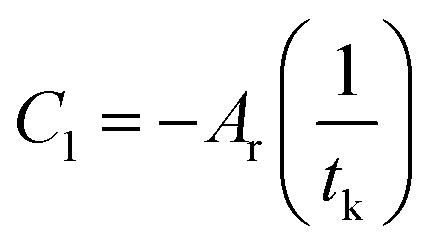
7
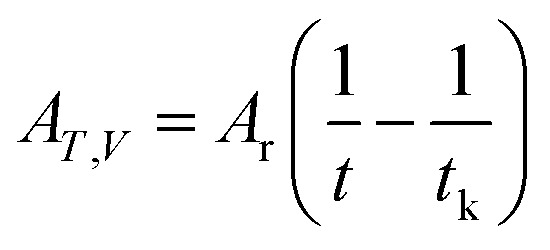


To correlate the parameters of the chemical affinity obtained from the experimental data with time, the two sides of eqn (7) are integrated separately:8
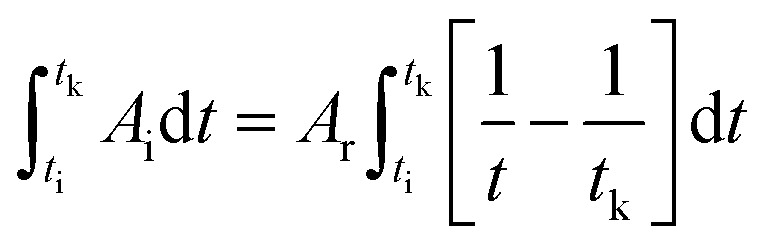


When the reaction reaches equilibrium (*t*_k_), the affinity decay rate is 0, so9
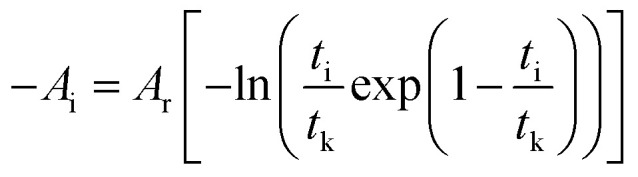


Eqn (9) is divided on both sides by (−*RT*), then10
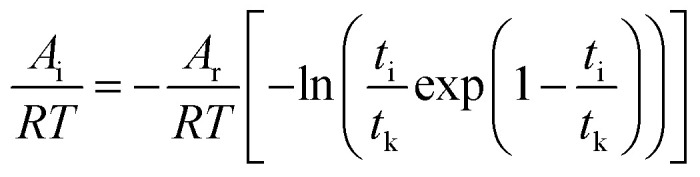


The degree of reaction as defined by reaction time is as follows.11
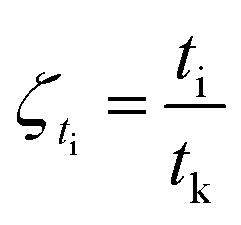


Gas hydrate formation refers to the process in which solute molecules (gas molecules) and water molecules act to form hydrated molecules. The change in the driving force *A*_i_ during hydrate formation is related to gas consumption. Thus, the degree of hydrate formation can be expressed by the consumption of gas in the process of hydrate formation instead of activity. For hydrate formation, formula (2) can be written as follows:12
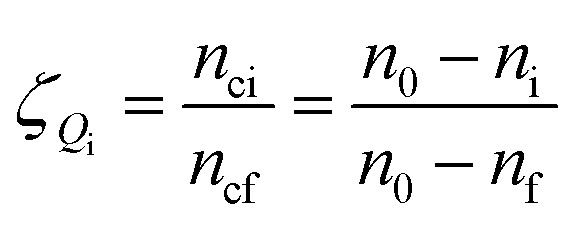
13
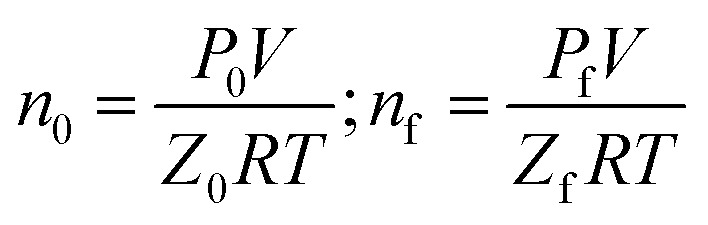


The subscript 0 refers to the initial state and f is the equilibrium state. *P* is the system pressure; *Z* is the gas compression factor; *n*_ci_ and *n*_cf_ are the numbers of moles of gas consumed at the reaction time *t*_i_ and at equilibrium respectively; *n*_0_, *n*_i_, and *n*_f_ represent the numbers of moles of gas in the initial state and at the times *t*_i_ and *t*_k_ of the reaction, respectively. From eqn (12) and (13),14
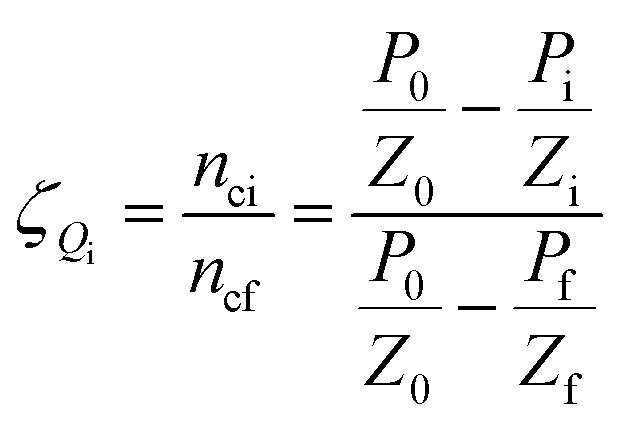


Eqn (2) and (14) give15
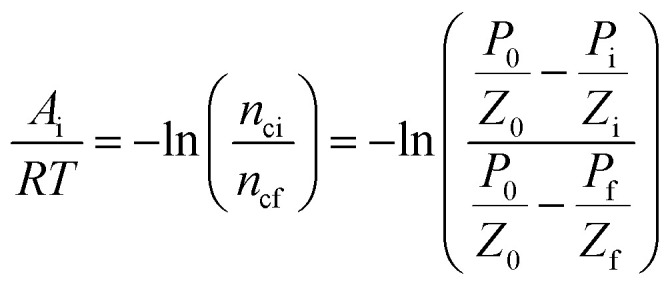


Eqn (10) and (15) give16
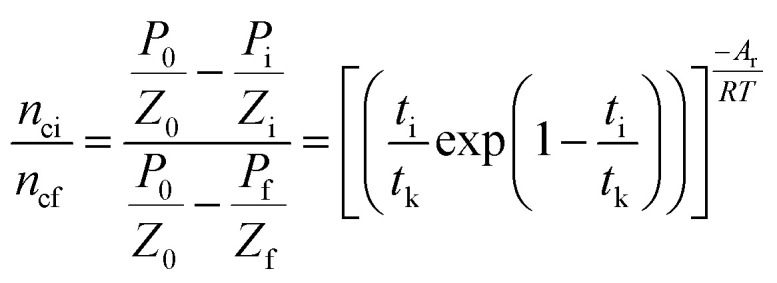


Taking the logarithm of both sides of eqn (16) gives17
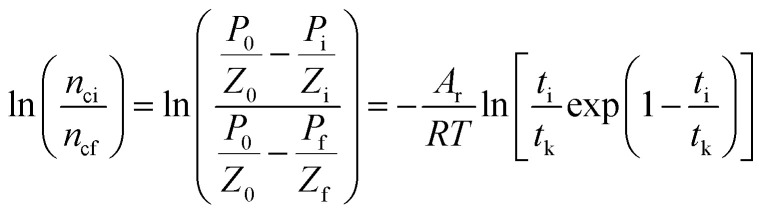
which is:
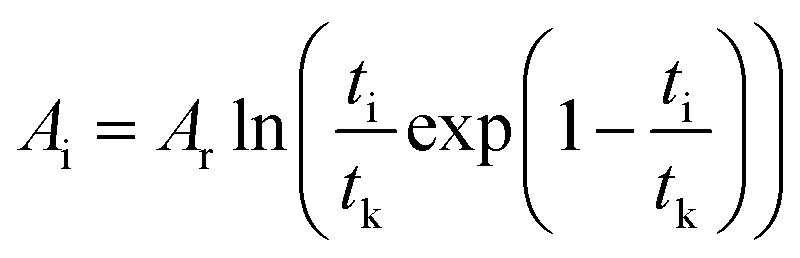
 as in eqn (9).

In the formula: *t*_k_ and 
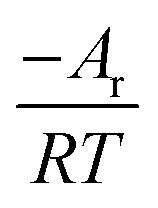
 are the kinetic parameters of the chemical affinity model. The absolute value of the slope
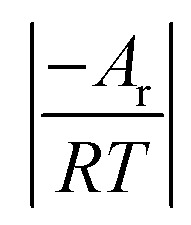
 can be a normalized rate constant, so the value of this slope can be employed for investigating the effects of parameters such as initial pressure, temperature, and additives on the hydrate formation kinetics.

By plotting the experimental data of hydrate formation, a plot of *A*_i_/*RT* and −ln[*t*_i_/*t*_k_exp(*t*_i_/*t*_k_)] can be made, and a straight line with zero intercept must be obtained. The slope of the line is −*A*_r_/*RT*, from which the values of the model parameters *t*_k_ and *A*_r_ can be obtained. The gas consumption amount *n*_ci_ in the hydrate formation process is predicted by formula (16).

**Fig. 2:**
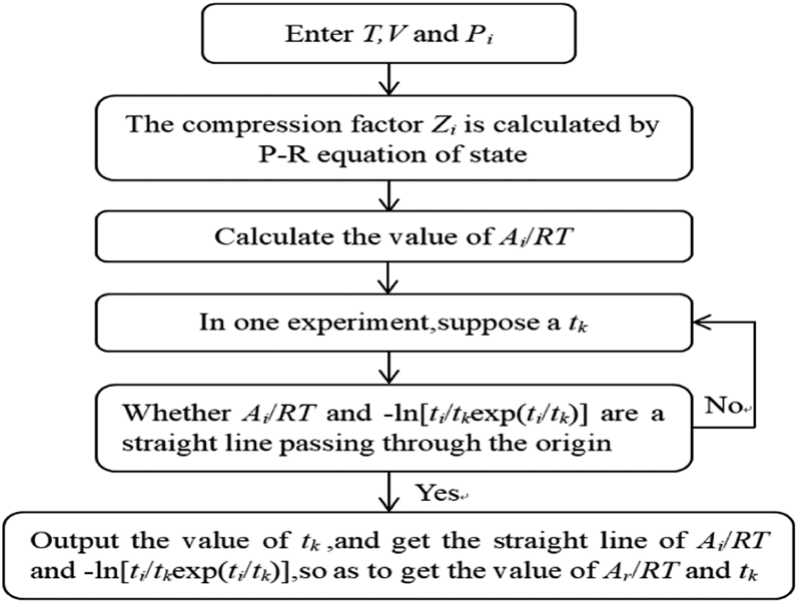
The algorithm for the calculation of the parameters of the chemical affinity model.

The model parameter calculation logic relationship is shown in Fig. 2.

Using Matlab software, according to the logical relationship of Fig. 2, the gas compression factor *Z*_i_, gas consumption *n*_ci_, reaction equilibrium time *t*_k_, kinetic parameters *A*_r_/*RT*, *etc.* were programmed.

## 4 Conclusion and analysis

### 4.1 The influence of the GO and SDS complex concentration on gas consumption

**Fig. 3:**
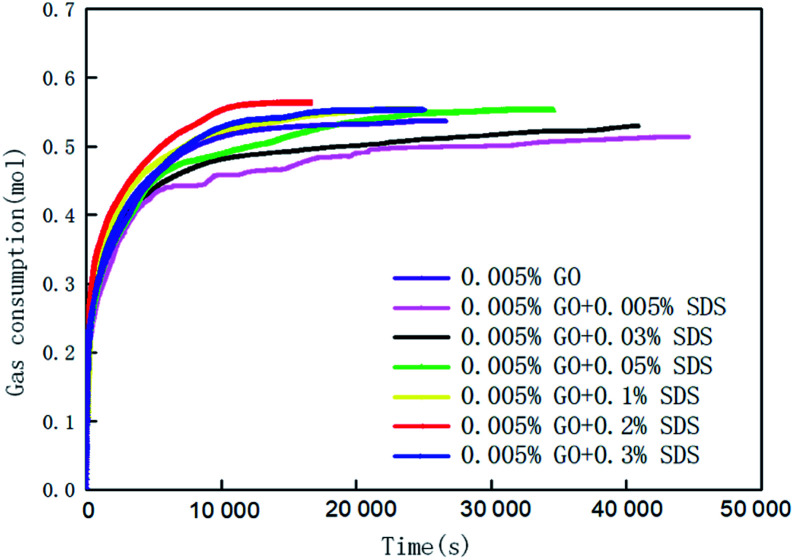
The change curves of gas consumption *versus* time in systems with 0.005% GO compounded with different concentrations of SDS.

**Fig. 4:**
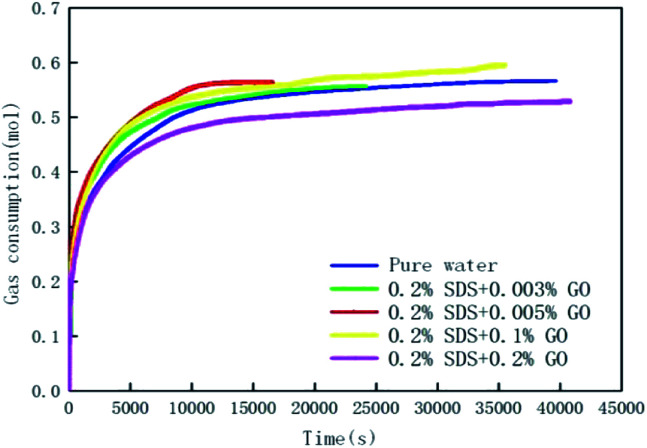
The change curves of gas consumption *versus* time in the pure water system, 0.2% GO compounded with different concentrations of GO systems.

Fig. 3 and 4 show the changes in gas consumption in GO and SDS compound solutions with different concentrations at 281.15 K, 4 MPa and 450 rpm. Fig. 3 and 4 show that the gas consumption rate tends to gradually decrease and to be flat. This is mainly because both SDS and GO can reduce the surface tension of water and further increase the gas–liquid contact area under the action of stirring. The gas can be better dissolved in water, provide more nucleation sites, and promptly release the latent heat in the hydration reaction, so that the rate of hydrate formation is accelerated. As the hydration reaction continues, the pressure in the kettle gradually decreases, the driving force decreases, and the gas consumption rate becomes slow until the phase equilibrium is reached. The results show that in the range of 0–0.2% SDS concentration, as the concentration of SDS increases, the CO_2_ gas consumption rate will increase and the gas consumption will gradually increase. This is mainly because a moderate concentration can enhance the dissolution of CO_2_, thereby accelerating the production rate and increasing the gas storage density. When the concentration of SDS in the 0.005% GO compound system reached 0.2%, the gas consumption reached the maximum value (0.5639 mol), which was 11.24% and 3.2% higher than that of pure water and a single 0.005% GO system, respectively. As the concentration continues to increase, the gas consumption decreases; this indicates that there is an optimum value for the concentration of SDS in the 0.005% GO compound system, which is about 0.2%. Fig. 4 shows that when GO is less than 0.005%, the gas consumption is increased but when the concentration is higher than 0.005%, the gas consumption is decreased. This is mainly because both SDS and GO can reduce the surface tension during the hydration process, thereby reducing the heat and mass transfer resistance and increasing the gas consumption. The results indicate that there is an optimum compounding concentration of about 0.005% GO + 0.2% SDS. At this time, its function as a surfactant can be fully utilized, and the rate of hydrate formation and gas storage can also be maximized.

### 4.2 The influence of the GO and SDS compound concentration on the generation time

**Fig. 5:**
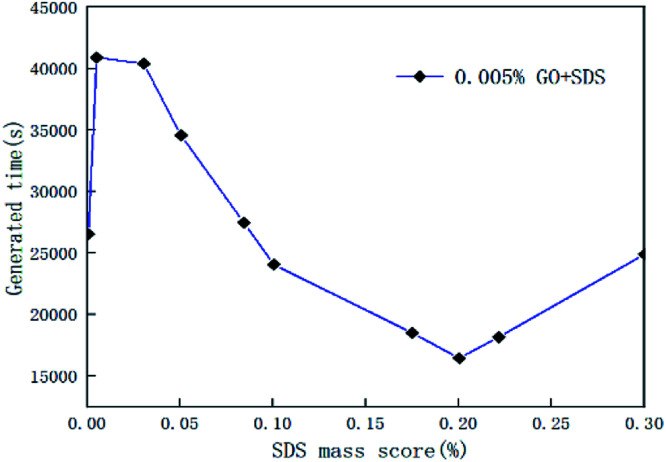
Formation time of CO_2_ hydrate with 0.005% GO and different concentrations of SDS systems.

**Fig. 6:**
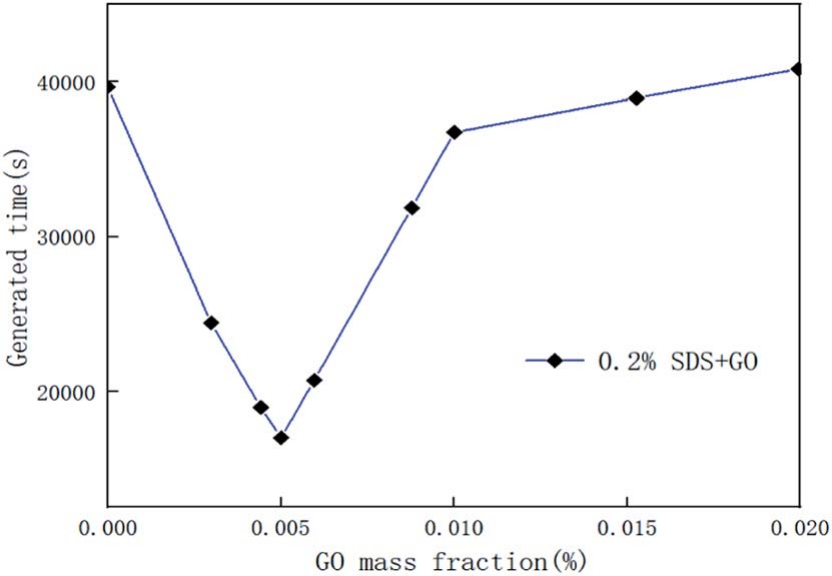
Formation time of CO_2_ hydrate in the pure water system, and systems with 0.2% SDS and different concentrations of GO.

In the course of the hydration reaction, when the system pressure is almost constant within 30 min, it is regarded as the end of the reaction. The hydrate formation time is defined as the time from the start to the end of the reaction. Fig. 5 is a graph of the reaction time of the compound system with 0.005% GO in different concentrations of SDS. Fig. 6 is a graph showing the formation time of the CO_2_ hydrate in pure water and the compound system with 0.02% SDS and different concentrations of GO. From Fig. 5 and 6, it is considered that the generation time first decreases and then increases with the increase in additives in the compound system. This is mainly because the proper concentration can enhance the heat and mass transfer efficiency and make the GO distribution uniform and form more nucleation points, speed up the reaction rate and reduce the generation time. However, when the concentration increased to a certain value, the generation time increased. It was seen from the experiment that this is mainly because when the concentration of the compound system reaches a certain level, agglomeration occurs, the heat and mass transfer efficiency is lowered, the amount of gas of participating in the hydration reaction is reduced, the reaction rate is decreased, and the reaction time is increased. Therefore, there is an optimal concentration for the compound of GO and SDS. The analysis considers that the optimum concentration is about 0.005% GO and 0.2% SDS. At this concentration, compared with pure water and a single 0.005% GO system, the generation time was shortened by 38.50% and 58.12% respectively.

## 5 Chemical affinity model analysis

### 5.1 The influence of the model parameters on the compound from GO and SDS

By calculation, under the conditions of 281.15 K, 4 MPa and 450 rpm, the chemical affinity kinetic model parameters in the GO and SDS compounded systems with different mass fractions are shown in Table 2. It can be seen from the table that the compound solution made from GO and SDS can accelerate the reaction to reach equilibrium; the promotion effect of the compound system with 0.005% GO and 0.2% SDS is more obvious. In the compound system with 0.005% GO and 0.2% SDS, the rate of hydrate reaching equilibrium is was the fastest (*t*_k_ = 10 000 s), and the promoting effect was the most obvious (−*A*_r_/*RT* = 0.2489). As the concentration of GO and SDS increased, the equilibrium pressure followed the trend of first increasing and then decreasing, and in the system with 0.005% GO + 0.2% SDS, the equilibrium pressure (*P*_f_) was the lowest (about 1.8549 MPa). This is consistent with the previous experimental results. As a kind of high-efficiency surfactant, SDS can reduce the interfacial tension and change the hydrophilic and lipophilic properties of the interface. After proper ultrasonic vibration, a uniformly dispersed GO solution was obtained, and the interlayer spacing was reduced so that the interlayer van der Waals forces were reduced. The GO nanoparticles were more uniformly dispersed in the solution, remained stable, were less prone to agglomeration, and fully exerted the foaming and wetting properties of the surfactant. GO has a large surface area, the heat and mass transfer efficiency is fast, and the surface is randomly distributed with hydroxyl and epoxy groups; the carboxyl and carbonyl groups were introduced at the edges and so it is both hydrophilic and hydrophobic.^24^ The hydrophobic group makes it easier for CO_2_ to enter into the solution, and the hydrophilic group forms a hydrogen bond with water, increasing the probability of forming a hydrate.

**Table 2:** The effects of GO compounded with SDS on the kinetic parameters of the chemical affinity model

Concentration (wt%)		Model parameters		Pressure (MPa)	
SDS	GO	*t*_k_ (s)	−*A*_r_/*RT*	*P*_0_	*P*_f_
0	0	33 013	0.2126	4	2.1967
0	0.005	11 385	0.2227	4	2.1115
0.005	0.005	11 337	0.2325	4	2.0789
0.03	0.005	11 315	0.2359	4	2.0891
0.05	0.005	11 190	0.2249	4	1.9961
0.1	0.005	10 088	0.2210	4	1.9563
0.2	0.005	10 000	0.2489	4	1.9489
0.3	0.005	10 061	0.2375	4	1.9521
0.2	0.003	10 291	0.2231	4	1.9563
0.2	0.01	11 296	0.2234	4	2.0372
0.2	0.02	11 356	0.2221	4	2.0325

### 5.2 The influence of pressure on model parameters

**Fig. 7:**
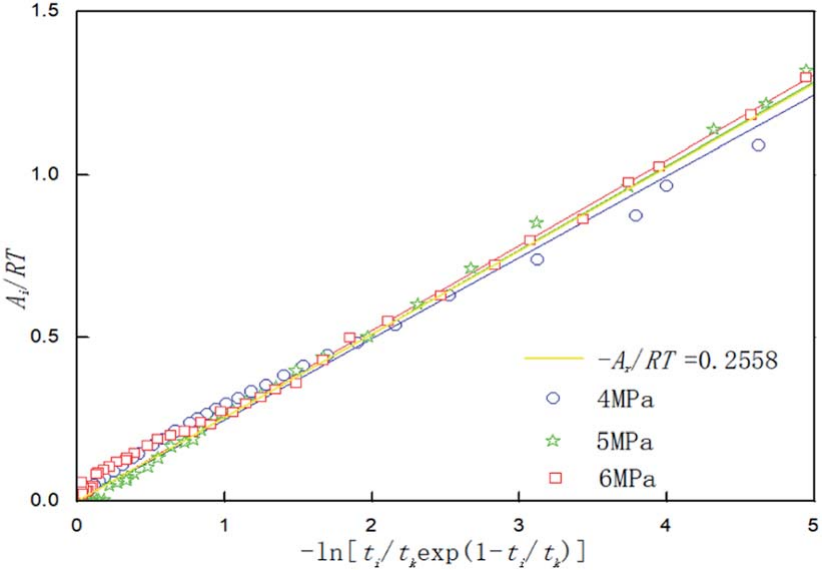
Affinity variation *versus* −ln[*t*_i_/*t*_k_exp(1 − *t*_i_/*t*_k_)] in the system with 0.005% GO and 0.2% SDS at 281.15 K,450 rpm and different initial pressures.

Table 3 shows the chemical affinity model parameters of different initial pressures (*P*_0_) at 281.15 K, 450 rpm, and the optimum composite accelerator mass fraction (0.005% GO + 0.2% SDS). It can be seen from the table that the initial pressure hardly affects the pressure at equilibrium (*P*_f_), but as the initial pressure increased, the reaction was accelerated and the time (*t*_k_) to reach equilibrium was reduced. This is mainly because the high initial pressure provides a large driving force (pressure) for the hydration reaction, the mass transfer resistance in the liquid phase is lowered, and the hydration reaction proceeds rapidly. Under different pressures, the value of the model parameter *A*_r_/*RT* changes only a little, and the average value is taken as the value (0.2558) of the kinetic model parameter |*A*_r_/*RT*| for predicting CO_2_ hydrate formation under this condition. Under the same conditions, Fig. 7 shows the fitting curves of the model and experimental parameters at different initial pressures and straight lines were obtained when |*A*_r_/*RT*| was 0.2558. It can be seen from Fig. 7 that the experimental and model parameters have the highest fitting degree at 6 MPa, which indicates that the larger the initial pressure, the higher the accuracy of the model. However, at different initial pressures, the model curve shows little difference, which indicates that the initial pressure has little effect on the model parameters.

**Table 3:** The effects of different initial pressures on kinetic parameters of chemical affinity model

Temperature (K)	*P*_0_ (MPa)	*P*_f_ (MPa)	Model parameters	
			*t*_k_ (s)	−*A*_r_/*RT*
281.15	4	1.9489	10 000	0.2489
281.15	5	1.9511	9764	0.2571
281.15	6	1.9589	9218	0.2614

### 5.3 Influence of temperature on model parameters

**Fig. 8:**
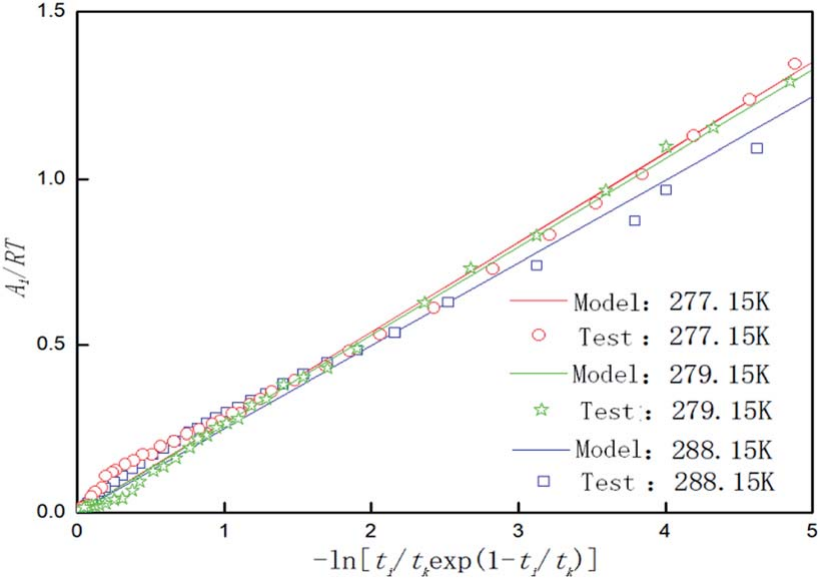
Affinity variation *versus* −ln[*t*_i_/*t*_k_exp(1 − *t*_i_/*t*_k_)] for CO_2_ hydrate formation in the system with 0.005% GO and 0.2% SDS at 4 MPa, 450 rpm and different temperatures.

Temperature is an important factor in the hydrate formation process, so the effect of temperature on the parameters in the model was studied separately in the model. Table 4 shows the chemical affinity model parameters for the 0.005% GO + 0.2% SDS compound system and different temperatures. Fig. 8 is the fitting curve of the experimental and model parameters under the same working conditions. As can be seen from Table 4 and Fig. 8, *P*_f_ is significantly different at different temperatures. The cooler the system gets, the lower the *P*_f_, the shorter the *t*_k_, and the larger the value of the model parameter (|*A*_r_/*RT*|). This indicates that the lower the temperature, the easier it is for the CO_2_ hydrate to be formed, the shorter the reaction time, and the faster the formation rate. This is mainly because the lower temperature provides a greater driving force for the reaction, the heat transfer resistance in the system becomes smaller, the reaction is more likely to occur and it proceeds rapidly; this conforms to the formation law of hydrates. With the increase from 277.15 K to 279.15 K, the equilibrium pressure increased by about 0.1171 MPa; with the increase from 279.15 K to 281.15 K, the equilibrium pressure increased by about 0.0775 MPa and there was a decrease in the growth. This shows that as the temperature increases, the influence on the equilibrium pressure is weakened. This may be because the temperature continues to increase and the hydrate formation conditions are not achieved. The experimental results were in good agreement with the model parameters.

**Table 4:** The effects of temperature on the kinetic parameters of the chemical affinity model for CO_2_ hydrate formation in the 0.005% GO + 0.2% SDS system

Temperature (K)	*P*_0_ (MPa)	*P*_f_ (MPa)	Model parameters	
			*t*_k_ (s)	−*A*_r_/*RT*
277.15	4	1.7543	8869	0.2698
279.15	4	1.8714	9632	0.2653
281.15	4	1.9489	10 000	0.2489

### 5.4 Model analysis of the promoting effect of different additives

**Fig. 9:**
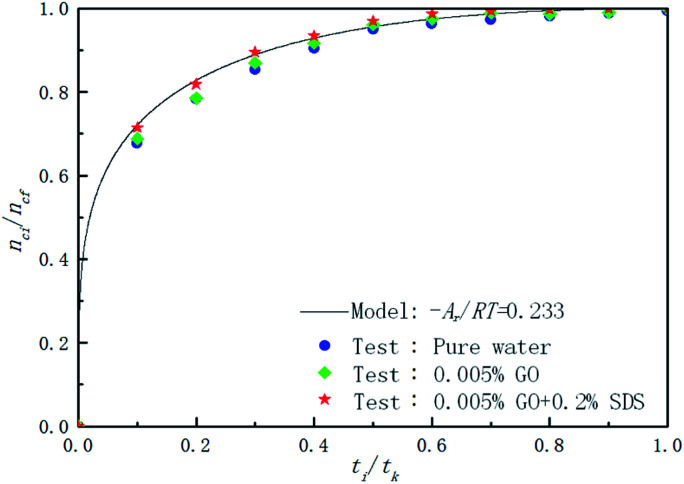
Comparison of the calculated and experimental normalized gas consumption in the pure water, 0.005% GO and 0.005% GO + 0.2% SDS systems.

**Fig. 10:**
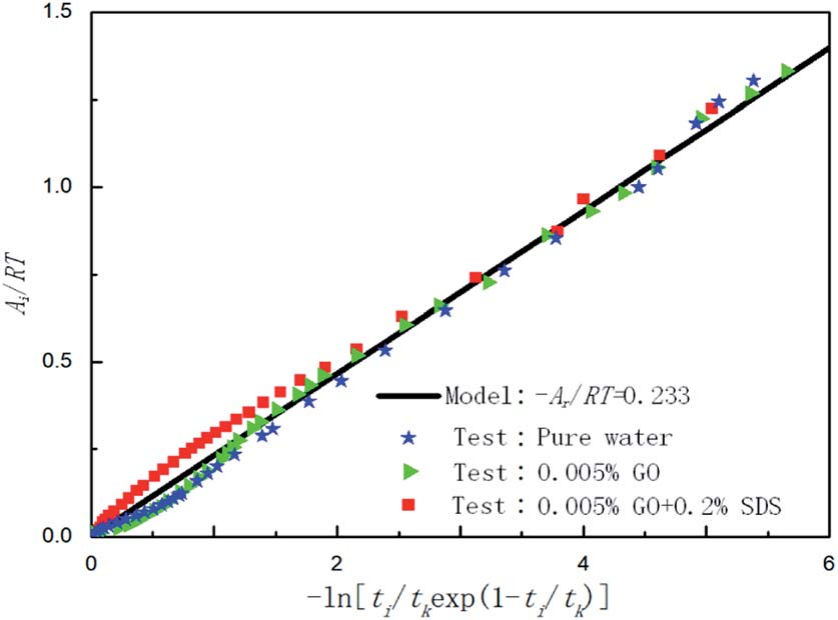
Affinity variation *versus* −ln[*t*_i_/*t*_k_exp(1 − *t*_i_/*t*_k_)] for CO_2_ hydrate formation in the systems with pure water, 0.005% GO and 0.005% GO + 0.2% SDS.

Fig. 9 shows the effect of pure water, 0.005% GO and 0.005% GO + 0.2% SDS reagent on the formation rate of CO_2_ hydrate at 281.5 K, 4 MPa and 450 rpm. As can be seen from the figure, in the 0.005% GO + 0.2% SDS system, the normalized gas consumption was the largest and the generation rate was the fastest; the second was 0.005% GO, and the formation rate in pure water was the slowest. This indicates that the promotion accelerator of 0.005% GO + 0.2% SDS is better than the single 0.005% GO. It can be seen from Table 2 that *A*_r_/*RT* obtained under the same working condition is almost constant. To make the model widely applicable, the same slope was taken under the same working conditions (−*A*_r_/*RT* = 0.233) to predict and study the formation of hydrates. Fig. 10 is a graph showing the chemical affinity model in pure water, 0.005% GO and 0.005% GO + 0.2% SDS reagent under the same conditions. Studies have shown that the calculation of the chemical affinity model under different additive systems was in good agreement with experimentally obtained parameters.

## 6 Model accuracy analysis

**Fig. 11:**
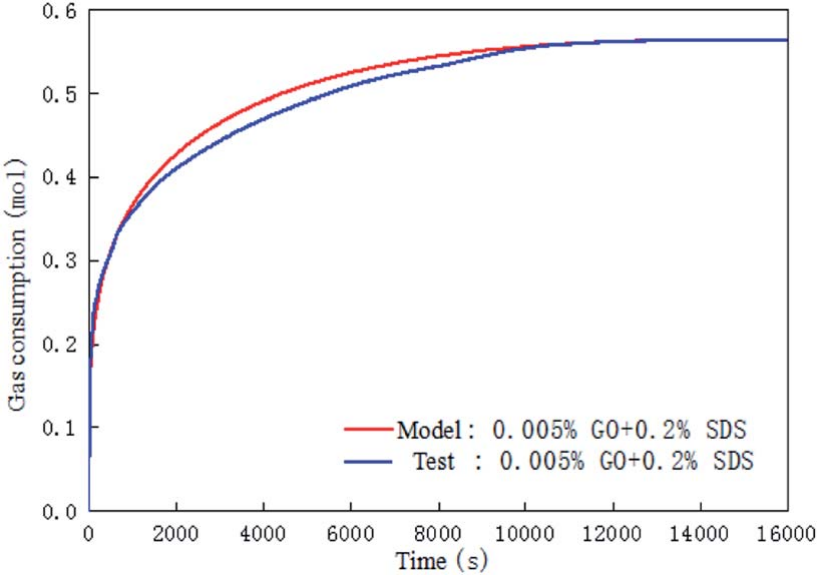
Comparison of the calculated and the experimental gas consumption in the system with 0.005% GO and 0.2% SDS.

Fig. 11 is a comparison of the experimental data with the data calculated by the chemical affinity model in the 0.005% GO + 0.2% SDS compound system at 281.15 K, 4 MPa, 450 rpm. The abscissa is the reaction time and the ordinate is gas consumption; the accuracy of the model prediction was checked by comparison of the gas consumption. It can be seen from the figure that in the initial stage of the reaction (0–2000 s), the model prediction data was consistent with the experimental data. As the reaction progressed, in the middle of the reaction (2000–10 000 s), the model prediction data was slightly larger than the experimental data, and at the end of the reaction (after 10 000 s), the model prediction data was consistent with the experimental data. Overall, the comparison shows that the data obtained by the model was in good agreement with the experimental data and can be used for the prediction of hydrate formation. It can be seen from Table 2 that *A*_r_/*RT* obtained under the same working condition was almost constant. To make the model widely applicable, the same slope was taken under the same working condition (−*A*_r_/*RT* = 0.233) to predict and study the CO_2_ hydrate formation.

## 7 Conclusion

(1) The promoting effect of CO_2_ hydrate formation on the complexation of GO + SDS was studied *via* experiments. The results showed that there was an obvious promoting effect. The composition ratio and mass fraction of the complex system had a significant influence on hydrate formation and obtained the best promotion mass fraction of 0.005% GO + 0.2% SDS. Compared with pure water and a single 0.005% GO system, the hydrate formation time was shortened by 69.7% and 12.2%, respectively, and the gas consumption was increased by 11.24% and 3.2%.

(2) The combination of SDS and GO caused the heat and mass transfer characteristics of the system to be mutually reinforced, which was beneficial to the rapid formation of the hydrate. GO was more evenly distributed in the solution containing SDS, providing more nucleation points and enhancing the ability to capture the gas. Therefore, the system temperature was fast and stable, the pressure drop was more obvious, the gas consumption was greatly increased, and the generation time was significantly shortened.

(3) The chemical affinity model for hydrate formation in the GO and SDS complex system was established. The model was programmed by Matlab and compared with the experimental results. The model analysis indicated that the concentration of the composite accelerator had a great influence on the model parameters *t*_k_, *A*_r_/*RT*, and the phase equilibrium pressure, and the optimal concentration value could be predicted, which was consistent with the experimental results. As the initial pressure increased and the temperature decreased, the time for the hydration reaction to reach equilibrium was shortened.

(4) The chemical affinity model is relatively simple, easy to solve, and is in good agreement with the experimental results. It can accurately predict the formation of hydrates in the compound system containing GO/SDS and is a good reference for the prediction of hydrate formation in other accelerator systems.

## Nomenclature

*A*_i_
: Affinity at state i
*A*_r_
: Proportionality constant
*t*_i_
: Time it takes to get to state i, s
*t*_k_
: Time it takes to get to k, s
*n*_ci_
: Number of moles of gas consumed at *t*_i_, mol
*n*_cf_
: Total number of moles of gas consumed, mol
R
: Ideal gas constant
T
: Temperature, K
P
: Pressure, Pa
Z
: Gas compression factor

### Subscripts

0: Initial state
i: State at time i
f: State of equilibrium

## Conflicts of interest

There are no conflicts to declare.

## Supplementary Material
